# Supplementary material for: The Small RNA Universe of Capitella teleta
Source: Front Mol Biosci. 2022 Feb 25;9:802814. doi: 10.3389/fmolb.2022.802814 (PMC8915122; doi:10.3389/fmolb.2022.802814)
Supplement: Supplementary file 1 [file DataSheet1.ZIP › Supplement/known/CAPTEscaffold_933_30844.pdf]

Provisional ID : CAPTEscaffold_933_30844
Score total : 31890.6
Score for star read(s) : 3.9
Score for read counts : 31880.5
Score for mfe : 1.6
Score for randfold : 1.6
Score for cons. seed : 3
Total read count : 62544
Mature read count : 58165
Loop read count : 0
Star read count : 4379

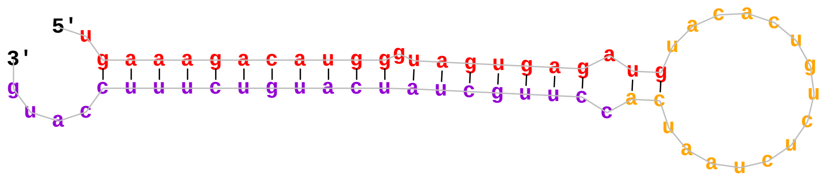

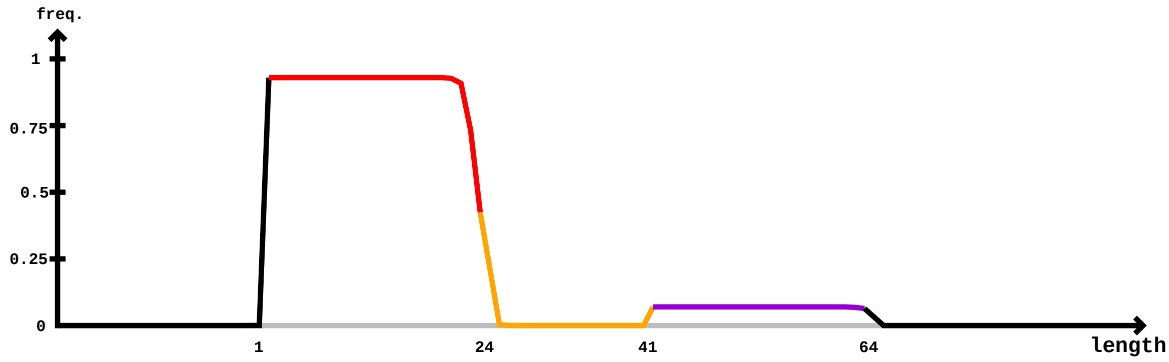


```
                    Mature                                   Star
5'- gacaauacagaggagcuccaugaaagacauggguagugagauguacacugucucuaaaucaccuugcuaucaugucuuuccauggagaacucuuaucgauagucguuuggcu  -3'  obs
    gacaauacagaggagcuccaugaaagacauggguagugagauguacacugucucuaaaucaccuugcuaucaugucuuuccauggagaacucuuaucgauagucguuuggcu       exp
    gacaauacagaggagcuccaugaaagacauggguagugagauguacacugucucuaaaucaccuugcuaucaugucuuuccauggagaacucuuaucgauagucguuuggcu       known
    (((.((....((((((((((((((((((((((.(((((((.((................)).))))))))))))))))).)))))))..)))))....)).)))........  reads  mm  sample

    ..caauacagaggagcucca............................................................................................  3      0   seq
    ..................Aaugaaagacauggguagugaga.......................................................................  1      1   seq
    ...................Cugaaagacauggguaguga.........................................................................  1      1   seq
    ...................augaaagacauggguagugaga.......................................................................  4      0   seq
    ...................Cugaaagacauggguagugagau......................................................................  1      1   seq
    ....................ugaaagacauggguagug..........................................................................  2      0   seq
    ....................ugaaagacauggguagAg..........................................................................  1      1   seq
    ....................ugaaagacauggguagugU.........................................................................  2      1   seq
    ....................ugaaagacauggguaguga.........................................................................  173    0   seq
    ....................ugaaagacauggguagugG.........................................................................  19     1   seq
    ....................ugaaagacauggUuaguga.........................................................................  1      1   seq
    ....................ugaaagacauggguagAga.........................................................................  1      1   seq
    ....................ugaaagacauggguagCgag........................................................................  1      1   seq
    ....................ugaaagacaugUguagugag........................................................................  1      1   seq
    ....................ugaaagacauggguaguAag........................................................................  1      1   seq
    ....................Agaaagacauggguagugag........................................................................  9      1   seq
    ....................uAaaagacauggguagugag........................................................................  2      1   seq
    ....................ugaaagacauggguagugaA........................................................................  6      1   seq
    ....................ugaGagacauggguagugag........................................................................  1      1   seq
    ....................ugaaagacauggguagAgag........................................................................  2      1   seq
    ....................ugaaagacaugAguagugag........................................................................  3      1   seq
    ....................Ggaaagacauggguagugag........................................................................  1      1   seq
    ....................ugaaagacauggguagugag........................................................................  1131   0   seq
    ....................ugaaagacauggguagugaU........................................................................  1      1   seq
    ....................ugaaagacauggguagugaga.......................................................................  10736  0   seq
    ....................ugaaagacauggguagugagU.......................................................................  16     1   seq
    ....................uCaaagacauggguagugaga.......................................................................  3      1   seq
    ....................Cgaaagacauggguagugaga.......................................................................  4      1   seq
    ....................ugaaagacauggguaguUaga.......................................................................  1      1   seq
    ....................ugaaagacauggguagGgaga.......................................................................  1      1   seq
    ....................ugaaagacauggguagugaAa.......................................................................  3      1   seq
```

Mature Star

gacaauacagaggagcuccaugaaagacauggguagugagauguacacugucucuaaucaccuugcuaucaugucuuuccauggagaacucuuaucgauagucguuuggcu			
....................ugaaagacaCggguagugaga..	2	1	seq
....................ugaaagUcauggguagugaga..	3	1	seq
....................ugaaagacauggguagugagG..	11	1	seq
....................ugaaagacaugAguagugaga..	8	1	seq
....................ugaaagacauggguaguAaga..	17	1	seq
....................ugaaGgacauggguagugaga..	3	1	seq
....................ugaaagacauggguagAgaga..	11	1	seq
....................ugaaagacaAggguagugaga..	14	1	seq
....................ugaaagacauggguaUugaga..	2	1	seq
....................Agaaagacauggguagugaga..	60	1	seq
....................ugaaagacauggguUgugaga..	2	1	seq
....................ugaaagacauggguaAugaga..	1	1	seq
....................ugUaagacauggguagugaga..	1	1	seq
....................Ngaaagacauggguagugaga..	1	1	seq
....................uAaaagacauggguagugaga..	15	1	seq
....................ugaaagGcauggguagugaga..	2	1	seq
....................ugaaagacauggguagugagC..	2	1	seq
....................ugaaagacauUgguagugaga..	1	1	seq
....................ugaaagacauggguagugaCa..	1	1	seq
....................ugaaagacauggAuagugaga..	1	1	seq
....................ugaaagacauggUuagugaga..	3	1	seq
....................Ggaaagacauggguagugaga..	6	1	seq
....................ugaaagacauggguagugGga..	1	1	seq
....................ugaaagacaGggguagugaga..	1	1	seq
....................ugaUagacauggguagugaga..	1	1	seq
....................ugaaaUacauggguagugaga..	1	1	seq
....................ugaaagacUuggguagugaga..	1	1	seq
....................ugaaagacaugUguagugaga..	1	1	seq
....................ugaaagacauAgguagugaga..	20	1	seq
....................ugaaagacauCgguagugaga..	2	1	seq
....................ugaaagacaugggAagugaga..	2	1	seq
....................ugaaagacGuggguagugaga..	3	1	seq
....................ugaaagaGauggguagugaga..	2	1	seq
....................ugaaCgacauggguagugaga..	1	1	seq
....................ugaaagaUauggguagugaga..	1	1	seq
....................ugaaagacauggguagugUga..	3	1	seq
....................ugaaagacaugCguagugaga..	1	1	seq
....................uUaaagacauggguagugaga..	4	1	seq
....................ugGaagacauggguagugaga..	3	1	seq
....................ugaaagacauggguaguCaga..	1	1	seq
....................ugaaagacauggguagugagaC...	3	1	seq
....................ugaaagGcauggguagugagau...	3	1	seq
....................ugaaGgacauggguagugagau...	4	1	seq
....................ugaaagaAauggguagugagau...	5	1	seq
....................ugaaagaGauggguagugagau...	3	1	seq
....................ugaaagacauggguagugagaG...	5	1	seq
....................ugaaagacauggguagugUgau...	4	1	seq
....................ugaaagacauggguagCgagau...	1	1	seq
....................Ggaaagacauggguagugagau...	17	1	seq
....................ugaaagacUuggguagugagau...	4	1	seq
....................ugaaagacauggguagugaAau...	1	1	seq
....................ugaaagacauggguUgugagau...	2	1	seq
....................ugaaagUcauggguagugagau...	1	1	seq
....................uAaaagacauggguagugagau...	39	1	seq
....................ugaaagacaugCguagugagau...	1	1	seq
....................ugaaagacauggguagugGgau...	6	1	seq
....................ugaGagacauggguagugagau...	5	1	seq
....................ugaaagacaCggguagugagau...	3	1	seq
....................ugaaagacaugggAagugagau...	9	1	seq
....................ugaaagacauggguGgugagau...	2	1	seq
....................ugaaagacauCgguagugagau...	3	1	seq
....................ugaaagacaAggguagugagau...	12	1	seq
....................ugaaaAacauggguagugagau...	3	1	seq
....................ugaaagacauggguagugaUau...	2	1	seq
....................ugaaagaUauggguagugagau...	3	1	seq
....................ugaaagacaugUguagugagau...	8	1	seq
....................Agaaagacauggguagugagau...	95	1	seq
....................ugaaaUacauggguagugagau...	2	1	seq
....................ugaaagacaugAguagugagau...	92	1	seq
....................ugaaagacauUgguagugagau...	2	1	seq

```
                    Mature                  Star
gacaauacagaggagcuccaugaaagacauggguagugagauguacacugucucuaaucaccuugcuaucaugucuuuccauggagaacucuuaucgauagucguuuggcu
....................uNaaagacauggguagugagau......................................................................  1      1  seq
....................ugaaagacauggCuagugagau......................................................................  2      1  seq
....................ugaaagacauggguagAgagau......................................................................  16     1  seq
....................Ngaaagacauggguagugagau......................................................................  2      1  seq
....................ugaaagacGuggguagugagau......................................................................  4      1  seq
....................ugaaaCacauggguagugagau......................................................................  2      1  seq
....................ugaaagacauggguagugagGu......................................................................  3      1  seq
....................ugaaagacauggguagugaCau......................................................................  1      1  seq
....................ugaaagacauggguagugagUu......................................................................  2      1  seq
....................uCaaagacauggguagugagau......................................................................  4      1  seq
....................uUaaagacauggguagugagau......................................................................  5      1  seq
....................ugaaagacauggguaguCagau......................................................................  2      1  seq
....................ugGaagacauggguagugagau......................................................................  1      1  seq
....................ugUaagacauggguagugagau......................................................................  2      1  seq
....................ugaaagacauggguaCugagau......................................................................  2      1  seq
....................ugaaagacauggguagugagau......................................................................  18664  0  seq
....................ugaaagacaugggCagugagau......................................................................  1      1  seq
....................ugaaagacauggguaAugagau......................................................................  1      1  seq
....................ugaaagacauggguagugagaA......................................................................  128    1  seq
....................ugaaagacauggguaUugagau......................................................................  2      1  seq
....................ugaaagacauggguaguAagau......................................................................  25     1  seq
....................Cgaaagacauggguagugagau......................................................................  1      1  seq
....................ugaaagacauAgguagugagau......................................................................  41     1  seq
....................ugaaagacauggguaguUagau......................................................................  2      1  seq
....................ugaaagacaGgguagugagau.......................................................................  1      1  seq
....................ugaaagacauggguagGgagaug.....................................................................  1      1  seq
....................uUaaagacauggguagugagaug.....................................................................  2      1  seq
....................ugaaagacauggAuagugagaug.....................................................................  4      1  seq
....................ugaaagacaCgguagugagaug......................................................................  2      1  seq
....................Cgaaagacauggguagugagaug.....................................................................  4      1  seq
....................ugaaagacauAgguagugagaug.....................................................................  40     1  seq
....................ugaaagacauggguagugagaCg.....................................................................  3      1  seq
....................ugaaagacaugggCagugagaug.....................................................................  2      1  seq
....................ugaaagacauggguagugagaug.....................................................................  21567  0  seq
....................ugaaagacauggCuagugagaug.....................................................................  3      1  seq
....................ugaaagacauggguagAgagaug.....................................................................  12     1  seq
....................uNaaagacauggguagugagaug.....................................................................  1      1  seq
....................ugaaagacauggguaguUagaug.....................................................................  2      1  seq
....................Agaaagacauggguagugagaug.....................................................................  111    1  seq
....................ugaaagacauggguagugagauC.....................................................................  8      1  seq
....................ugaaagaUauggguagugagaug.....................................................................  1      1  seq
....................ugaaagacaugUguagugagaug.....................................................................  5      1  seq
....................ugaaaCacauggguagugagaug.....................................................................  3      1  seq
....................ugaaagacauggguagugagauU.....................................................................  830    1  seq
....................ugaaagacauggguagugaAaug.....................................................................  3      1  seq
....................ugaaagaGauggguagugagaug.....................................................................  3      1  seq
....................ugaaGgacauggguagugagaug.....................................................................  6      1  seq
....................ugaaagacauggguaguCagaug.....................................................................  1      1  seq
....................ugaaagacauggUuagugagaug.....................................................................  6      1  seq
....................Ggaaagacauggguagugagaug.....................................................................  22     1  seq
....................ugaaagacauggguagugaUaug.....................................................................  1      1  seq
....................ugaaagacauggguaUugagaug.....................................................................  2      1  seq
....................ugaaagacauggguaguAagaug.....................................................................  33     1  seq
....................ugaaagacauggguagCgagaug.....................................................................  2      1  seq
....................ugaaagacauggguagugagaGg.....................................................................  3      1  seq
....................ugaaagacaugCguagugagaug.....................................................................  4      1  seq
....................ugaaagacGuggguagugagaug.....................................................................  4      1  seq
....................ugaaagacUuggguagugagaug.....................................................................  2      1  seq
....................Ngaaagacauggguagugagaug.....................................................................  8      1  seq
....................ugGaagacauggguagugagaug.....................................................................  2      1  seq
....................ugaaagacauggguagugagUug.....................................................................  1      1  seq
....................uCaaagacauggguagugagaug.....................................................................  5      1  seq
....................ugaaagacauCgguagugagaug.....................................................................  2      1  seq
....................ugaGagacauggguagugagaug.....................................................................  6      1  seq
....................ugaaagUcauggguagugagaug.....................................................................  1      1  seq
....................ugaaagacauUgguagugagaug.....................................................................  1      1  seq
....................ugaaagacaugggguGgugagaug....................................................................  1      1  seq
....................ugaUagacauggguagugagaug.....................................................................  1      1  seq
....................ugaaUgacauggguagugagaug.....................................................................  2      1  seq
....................ugaaagacaAgguagugagaug......................................................................  19     1  seq
```

```
                    Mature                                      Star
gacaauacagaggagcuccaugaaagacauggguagugagauguacacugucucuaaucaccuugcuaucaugucuuuccauggagaacucuuaucgauagucguuuggcu
....................ugaaaUacauggguagugagaug.................................................................... 2     1   seq
....................ugaaagacauggguagugagauA.................................................................... 257   1   seq
....................ugaaagacaugAguagugagaug.................................................................... 123   1   seq
....................ugaaagacauggguagugUgaug.................................................................... 5     1   seq
....................uAaaagacauggguagugagaug.................................................................... 38    1   seq
....................ugaaagGcauggguagugagaug.................................................................... 4     1   seq
....................ugaaagCcauggguagugagaug.................................................................... 1     1   seq
....................ugaaagacauggguagugagaAg.................................................................... 5     1   seq
....................ugaaagacauggguagugagGug.................................................................... 4     1   seq
....................ugaaagacauggguaAugagaug.................................................................... 3     1   seq
....................ugaaagacauggguagugGgaug.................................................................... 4     1   seq
....................ugaaagacaugggAagugagaug.................................................................... 4     1   seq
....................ugaaaAacauggguagugagaug.................................................................... 1     1   seq
....................ugUaagacauggguagugagaug.................................................................... 1     1   seq
....................ugaaaNacauggguagugagaug.................................................................... 1     1   seq
....................ugaaagaAauggguagugagaug.................................................................... 6     1   seq
....................Agaaagacauggguagugagaugu................................................................... 14    1   seq
....................ugaaagacauggguagugagaugu................................................................... 2362  0   seq
....................ugaaagacauggguagugagaAgu................................................................... 2     1   seq
....................ugaaagacauggguagugagaugC................................................................... 2     1   seq
....................ugaaagacauggguagugagauAu................................................................... 20    1   seq
....................ugaaagacaugggAagugagaugu................................................................... 1     1   seq
....................ugaaagacauggguagAgagaugu................................................................... 2     1   seq
....................ugaaagaAauggguagugagaugu................................................................... 1     1   seq
....................ugaaagacauggguagugagaCgu................................................................... 1     1   seq
....................ugaaagacauggguagugagaugA................................................................... 632   1   seq
....................ugaaagacauggguagugagauUu................................................................... 32    1   seq
....................ugaaagacaugggCagugagaugu................................................................... 1     1   seq
....................ugaaagacauggguagCgagaugu................................................................... 1     1   seq
....................ugaaagaUauggguagugagaugu................................................................... 1     1   seq
....................ugaaagacaGggguagugagaugu................................................................... 1     1   seq
....................ugaaagacaugAguagugagaugu................................................................... 1     1   seq
....................Ngaaagacauggguagugagaugu................................................................... 1     1   seq
....................ugaaagacaugggguUgugagaugu.................................................................. 2     1   seq
....................ugaaagacauCgguagugagaugu................................................................... 1     1   seq
....................ugaaagacauAgguagugagaugu................................................................... 5     1   seq
....................ugaaagacauggCuagugagaugu................................................................... 1     1   seq
....................ugaGagacauggguagugagaugu................................................................... 1     1   seq
....................Ggaaagacauggguagugagaugu................................................................... 1     1   seq
....................ugaaagacauggguaAugagaugu................................................................... 1     1   seq
....................ugaaagacauggAuagugagaugu................................................................... 1     1   seq
....................ugaaagacauggguagugagaugG................................................................... 11    1   seq
....................ugaaagacauggguaguAagaugu................................................................... 3     1   seq
....................ugGaagacauggguagugagaugu................................................................... 1     1   seq
....................uAaaagacauggguagugagaugu................................................................... 10    1   seq
....................ugaaagacauggguagugagaugua.................................................................. 8     0   seq
....................ugaaagacauggguagugagauguU.................................................................. 160   1   seq
....................ugaaagacauggguagugagaugAa.................................................................. 21    1   seq
....................ugaaagacauggguagugagauguaU................................................................. 18    1   seq
....................ugaaagacauggguagugagauguaA................................................................. 6     1   seq
....................ugaaagacauggguagugagaugAac................................................................. 2     1   seq
....................ugaaagacauggguagugagaugAaca................................................................ 2     1   seq
....................ugaaagacauggguagugagauguacacu.............................................................. 1     0   seq
....................ugaaagacauggguagugagauguacacugu............................................................ 2     0   seq
.....................gaaagacauggguagugaga...................................................................... 2     0   seq
.....................gaaagGcauggguagugagau..................................................................... 1     1   seq
.....................gaaagacauggguagugagau..................................................................... 10    0   seq
.....................gaaagacauggguagugagaug.................................................................... 9     0   seq
.....................Uaaagacauggguagugagaug.................................................................... 1     1   seq
.....................gaaagGcauggguagugagaug.................................................................... 2     1   seq
.....................gaaagacauggguagugagauU.................................................................... 1     1   seq
.....................gaaagacauggguagugagaugu................................................................... 1     0   seq
......................aaagacauggguagugaga...................................................................... 1     0   seq
......................aaagacauggguagugagaug.................................................................... 1     0   seq
......................aaagacauggguagugagaugu................................................................... 5     0   seq
............................................................ccuugcuaucaugucuuucc............................... 5     0   seq
............................................................ccuugcuaucaugucuuucca.............................. 109   0   seq
............................................................ccAugcuaucaugucuuuccau............................. 1     1   seq
............................................................ccuugcuaucaugucuuuccau............................. 247   0   seq
............................................................cUuugcuaucaugucuuuccau............................. 1     1   seq
```

```
                    Mature                                  Star
gacaauacagaggagcuccaugaaagacauggguagugagauguacacugucucuaaucaccuugcuaucaugucuuuccauggagaacucuuaucgauagucguuuggcu
............................................................ccuugcuaucaugucuuuccaA.............................   2     1   seq
............................................................ccuAgcuaucaugucuuuccau.............................   1     1   seq
............................................................ccuugAuaucaugucuuuccau.............................   1     1   seq
............................................................ccuuAcuaucaugucuuuccaug............................   2     1   seq
............................................................Ncuugcuaucaugucuuuccaug............................   1     1   seq
............................................................ccAugcuaucaugucuuuccaug............................   8     1   seq
............................................................ccuugcuaucaugucuuuAcaug............................   1     1   seq
............................................................Acuugcuaucaugucuuuccaug............................   1     1   seq
............................................................ccuugcuaucaugucuuuccUug............................   1     1   seq
............................................................Ucuugcuaucaugucuuuccaug............................   4     1   seq
............................................................ccuugcuaGcaugucuuuccaug............................   1     1   seq
............................................................ccuugAuaucaugucuuuccaug............................   3     1   seq
............................................................ccuugcuaucaugucAuuccaug............................   1     1   seq
............................................................ccuugcuaucaugucuuuccauU............................   21    1   seq
............................................................ccuugcuaucaugucuuuccNug............................   1     1   seq
............................................................ccuugcuaucaugucuAuccaug............................   4     1   seq
............................................................ccuugcuaucaugucuuuccaGg............................   2     1   seq
............................................................ccuugcuaucaugucuuuccaug............................   3881  0   seq
............................................................ccuugcuaucaAgucuuuccaug............................   1     1   seq
............................................................ccuugcuaucaugAcuuuccaug............................   2     1   seq
............................................................cAuugcuaucaugucuuuccaug............................   2     1   seq
............................................................ccuAgcuaucaugucuuuccaug............................   3     1   seq
............................................................ccuugcuaAcaugucuuuccaug............................   2     1   seq
............................................................ccuugcuaucaugucuuuccauA............................   7     1   seq
............................................................ccuugcAaucaugucuuuccaug............................   2     1   seq
............................................................ccuugcuaucaugucuuuCuaug............................   5     1   seq
............................................................ccuugGuaucaugucuuuccaug............................   1     1   seq
............................................................ccuugcuaucaugCcuuuccaug............................   2     1   seq
............................................................ccuugcuaucaugucuGuccaug............................   1     1   seq
............................................................ccuugcuaucaugucuuucAaug............................   2     1   seq
............................................................ccuugcuauUaugucuuuccaug............................   5     1   seq
............................................................ccuugcuaucaugucuuuUcaug............................   1     1   seq
............................................................ccuuUcuaucaugucuuuccaug............................   1     1   seq
............................................................ccuugcuaucaugucuuuccauC............................   1     1   seq
............................................................ccuugcuaucaugucuuucGaug............................   1     1   seq
............................................................ccuugcuaucaugucuuuccaugA...........................   1     1   seq
............................................................ccuugcuaucaugucuuuccaugU...........................   31    1   seq
.............................................................cCugcuaucaugucuuuccau.............................   1     1   seq
.............................................................cugcuaucaugucuuuccaug............................   6     0   seq
..............................................................uugcuaucaugucuuuccaug............................   1     0   seq
..............................................................uugcuaucaugucuuuccaugg...........................   1     0   seq
...................................................................................gagaacucuuaucgauagucguuA....   1     1   seq
...................................................................................gagaacucuuaucgauagucguuuggcu   1     0   seq
```
